# Experimental evolution of the grain of metabolic specialization in yeast

**DOI:** 10.1002/ece3.2151

**Published:** 2016-05-11

**Authors:** Pedram Samani, Graham Bell

**Affiliations:** ^1^ Biology DepartmentMcGill UniversityMontrealQCH3A 1B1Canada; ^2^ University of Montana32 Campus DriveMissoula59812Montana

**Keywords:** Cost of adaptation, functional interference, local adaptation, metabolic pathway, metabolic specialization, mutation accumulation, reciprocal transplant assay, *Saccharomyces paradoxus*, selection, trade‐off

## Abstract

Adaptation to any given environment may be accompanied by a cost in terms of reduced growth in the ancestral or some alternative environment. Ecologists explain the cost of adaptation through the concept of a trade‐off, by which gaining a new trait involves losing another trait. Two mechanisms have been invoked to explain the evolution of trade‐offs in ecological systems, mutational degradation, and functional interference. Mutational degradation occurs when a gene coding a specific trait is not under selection in the resident environment; therefore, it may be degraded through the accumulation of mutations that are neutral in the resident environment but deleterious in an alternative environment. Functional interference evolves if the gene or a set of genes have antagonistic effects in two or more ecologically different traits. Both mechanisms pertain to a situation where the selection and the alternative environments are ecologically different. To test this hypothesis, we conducted an experiment in which 12 experimental populations of wild yeast were each grown in a minimal medium supplemented with a single substrate. We chose 12 different carbon substrates that were metabolized through similar and different pathways in order to represent a wide range of ecological conditions. We found no evidence for trade‐offs between substrates on the same pathway. The indirect response of substrates on other pathways, however, was consistently negative, with little correlation between the direct and indirect responses. We conclude that the grain of specialization in this case is the metabolic pathway and that specialization appears to evolve through mutational degradation.

## Introduction

Economic and ecological communities are structured by the pattern of specialization. In economic communities, this constitutes the division of labor, reflecting how different kinds of employment are distributed among people. The analogous feature of ecological communities is the extent to which individuals are restricted to particular sites or diets, or resistant to particular parasites or predators. In either case, the ideal type is the perfect generalist who is capable of flourishing in all employments or thriving in all sites more successfully than any rival. In practice, this ideal state is seldom or never attained, at least for any considerable period of time (Kawecki 1994; Gilchrist 1995; Meijden 1996; Wei et al. 2015). In human societies, a division of labor is enforced by law, or emerges spontaneously from the increased productivity made possible by devoting exclusive attention to a narrow range of tasks. Among other organisms, the processes that favor specialization are different, but nonetheless invoke the greater fitness of specialists, and conversely the inferiority of generalists, over some part of the range of conditions available to the population. This constitutes a cost of adaptation: Enhanced performance in the ambient conditions of growth is associated with regress in the ancestral conditions, or in some defined alternative conditions of interest (Stearns 1989; Elena and Lenski 2003; Kawecki et al. 2012).

Ecologists explain the cost of adaptation and thus the evolution and maintenance of specialization, through the hypothesis, derived from economics, of a trade‐off, meaning that increased fitness over some more or less restricted range of conditions entails reduced fitness, relative to rivals, in conditions outside this range. There are two well‐understood causes of trade‐offs. The first is that alternative morphological structures, physiological processes, or behaviors may be incompatible. For example, a limb with the form of a hinged lever can produce either a slow, powerful stroke, or a rapid, weak stroke, depending on the position of the fulcrum relative to the point of application of force; speed and strength are inversely related by the laws of mechanics. Secondly, any feature that is never used will tend to deteriorate. This is because loss‐of‐function mutations in the genes which encode it are neutral so long as it does not contribute to fitness (Bell 2008). There are numerous examples of both functional interference (Cooper and Lenski 2000, 2010; Lunzer et al. 2002; Hietpas et al. 2013) and mutational degradation (Reboud and Bell 1997; MacLean and Bell 2002; Ostrowski et al. 2007; Behe 2010; Kvitek and Sherlock 2013) from experiments in which a population has been exposed to a novel environment.

Any population with access to several substitutable resources may evolve into a single broad generalist, or into a mixture of narrow specialists, or toward some intermediate position, depending on the trade‐offs between alternative competencies (Kassen 2002, 2009). Trade‐offs are more likely to occur when alternative resources require different and exclusive competencies, and are least likely to occur when they can be exploited in a similar manner with the same equipment. This suggests that they can be predicted by organizing resources hierarchically, from the most to the least inclusive categories. The most inclusive metabolic categories utilize resources so different that they require completely different cellular machineries, such as heterotrophic and photoautotrophic growth. Within such broad categories, there may be subsystems consisting of linked metabolic pathways, such as fermentation and respiration, that are incompatible because a given substrate molecule can be transformed through only one or the other. At a finer scale, there are individual metabolic pathways, such as glycolysis or the tricarboxylic acid (TCA) cycle. The finest scale of all consists of an individual substrate that is metabolized at some point along a given pathway. The leading ecological attribute of a population is the “grain of specialization” that represents its position on this hierarchy.

### Specialization among and within metabolic pathways

Natural populations of microbes encounter a diversity of potential substrates, metabolized through several different pathways, and may evolve a greater or lesser degree of specialization (Samani et al. 2015). The evolution of the grain of specialization can be studied experimentally by restricting the diet to a single substrate and measuring the growth of a lineage on this and on other substrates, on the same or on different metabolic pathways, in subsequent generations. The position of a target substrate on a metabolic pathway can be used to predict trade‐offs as the result of how functional interference and mutational degradation are expected to act on another substrate on the same or on a different pathway.

The general principle governing the degree of functional interference is that the more similar are two tasks the less likely they are to interfere with one another. Hence, adaptation to a target substrate, supplied as sole carbon source, is less likely to interfere with the utilization of another substrate on the same pathway and more likely to interfere with the utilization of a substrate on a different pathway.

Mutational degradation may affect any nontarget substrate. It is always likely to reduce growth on substrates belonging to another pathway if, as a result of the experimental treatment, this pathway is no longer active. Hence, growth on off‐pathway substrates is expected to be reduced as the indirect response to the exclusive provision of a single substrate. For substrates on the same pathway, the likelihood of degradation will depend on the position of the target substrate and the topology of the pathway. If a given substrate is supplied in excess of the rate at which it can be processed by the existing cellular machinery, the direct response to selection will be an improvement in this machinery through functional complementation, for example, by the modification of transport, regulatory, or catabolic pathways. Overall growth will depend on the flux along the pathway as a whole, however, which may be constrained by the capacity of downstream steps in the pathway, which may act as bottlenecks preventing any improvement from being fully implemented. Hence, according to Bell (2007), the next successful mutation will involve the substrate immediately upstream of the furthest downstream bottleneck. More generally, we expect the indirect response to be positive for substrates downstream of the target substrate. Upstream substrates, on the other hand, are no longer used, and the machinery responsible for their metabolism will eventually deteriorate through mutational degradation. This will be expressed as a negative indirect response for substrates upstream of the target substrate. For example, in the simple linear pathway A → B → C, the provision of B as the sole substrate is expected to lead to enhanced growth on C and reduced growth on A. In a branched pathway where metabolism may proceed from A to either B or C, adaptation to either downstream substrate may lead to loss of function for both the alternative and the upstream substrate. In a cyclical pathway, any given substrate can both be transformed into and generated from any other substrate, either directly or indirectly. The indirect response should therefore be positive for all substrates on the pathway.

In order to demonstrate a cost of adaptation, it must first be established that adaptation to a given substrate has in fact occurred, and then that growth on alternative substrates has been compromised. This is carried out most convincingly through a reciprocal transplant assay in which every line is assayed on every substrate. The results show whether there is interaction between selection and assay environments; whether there is a positive direct response to selection; and finally whether the direct response varies among replicate selection lines. If these conditions are satisfied, the cost (or benefit) of adaptation will be expressed through the indirect response to selection, which we predict will be:

1. negative for off‐pathway substrates through mutational degradation or functional interference;
2. negative for upstream substrates on the same pathway through mutational degradation;
3. positive for downstream substrates, including all substrates in a cyclical pathway, through functional complementation.

In this report, we shall describe an experiment in which populations of wild yeast, *Saccharomyces paradoxus*, were each grown on a single substrate, constituting the sole source of carbon and energy. The objective of the experiment was to estimate the grain of specialization that evolves when a population is restricted to a single defined substrate.

## Materials and Methods

### Ancestor

We used a single wild‐type strain of *S. paradoxus* collected from the Gault Nature Reserve of McGill University at Mont St‐Hilaire, Quebec, as the ancestor for this experiment.

### Substrates

We selected isogenic yeast populations on substrates belonging to three kinds of pathway; linear, cyclical, and branched. For each pathway, we chose three substrates, plus a fourth substrate belonging to a different pathway.

1. *Linear pathway*: Glycolysis is an example of a linear pathway. We chose raffinose (raf), fructose (fru), and pyruvate (pyr), as the three substrates located on this pathway: Raffinose is the furthest upstream substrate, fructose is intermediate, and pyruvate is downstream in the pathway. We chose aspartate (asp) as the fourth substrate: It is catabolized through the Krebs cycle after conversion to oxaloacetate.
2. *Branched pathway*: Glutamate (glu), proline (pro), and citrulline (cit) are catabolized through a complex and branched pathway to TCA cycle intermediary metabolites such as fumarate and ketoglutarate. We chose xylose (xyl) as the fourth substrate: It is converted to xylulose‐5‐phosphate and is then metabolized through the pentose phosphate pathway.
3. *Cyclical pathway*: Succinate (suc), fumarate (fum), and malate (mal) are the three substrates that participate in the TCA cycle to generate energy in aerobic metabolism. We used melibiose (mel) as the fourth substrate: It is hydrolyzed to glucose and galactose and subsequently metabolized through glycolysis.

Each set of four substrates was treated as a separate experiment in assays and analysis.

### Selection experiment

Selection lines were cultured in 12‐well plates containing minimal medium supplemented with 3% w/v of each of the carbon sources, using 12 replicates per treatment. Lines were transferred every 7 days and assayed and stored frozen every 10 transfers.

### Reciprocal transplant assay

Inoculation procedures were performed at the CIAN robotics/automation core facility (McGill University) on a Biomek FX liquid handler and an ORCA robotic arm controlled by the SAMI software (Beckman, Mississauga, ON, Canada). The response to selection was evaluated by a reciprocal transplant assay in which each selection line was grown in each of the four substrates chosen for a given pathway. The response variable was maximum yield (as optical density, OD), which corresponds with growth at transfer. Each pathway was evaluated by a separate assay. Each of the three assays comprised 4 selection environments ×4 assay environments ×12 replicate lines per selection environment ×2 replicate cultures of each line. In addition, we recorded the yield of the ancestor in all 12 assay environments. Two complete assays were conducted, the first after 24 cycles and the second after 42 cycles.

### Analysis of the reciprocal transplant assay

The direct and indirect responses to selection can be evaluated from a reciprocal transplant assay in which all evolved lines are grown on all substrates. This reciprocal transplant assay generates a matrix of scores for all combinations of selection environment and assay environment. If populations have become specifically adapted to the environment in which they were selected, scores along the leading diagonal of the matrix (in which the assay environment corresponds to the selection environment) will be consistently greater than scores in off‐diagonal cells. This will generate a statistical interaction between selection and assay environments that can be used to evaluate the occurrence of specific adaptation.

The results of the assay can then be used to answer two questions. The first is whether growth on a particular substrate is greater among lines that have been propagated on that substrate than among lines that have been propagated on other substrates. This expresses the improvement in a given assay environment that has been caused by natural selection in that environment, relative to any improvement that might be caused in that environment by selection in other environments. This is the sense in which “local adaptation” is usually understood (Kawecki and Ebert 2004). The second question is whether lines that have been propagated on a given substrate grow better on that substrate than on other substrates. This shows whether the improvement of a line in the environment of selection is consistently associated with its deterioration in other environments. This is often called the “cost of adaptation” (Levins 1968).

To evaluate the extent and the cost of adaptation, the overall response *R*(*ijk*) of the *k*‐th replicate line subjected to the *j*‐th selection treatment and grown in the *i*‐th assay conditions can be partitioned as follows:

R(ijk)−A(i)=[R(i..)−A(i)]+[R(ij.)−R(i..)]+[R(ijk)−R(ij.)],

where the subscript dots indicate averaging. The three components of the overall response are as follows.

1. The first term on the right‐hand side is the general response associated with a given assay environment, estimated as the difference between the average of all selection treatments in a given assay environment, *R*(*i*..), and the growth of the ancestor in that environment, *A*(*i*). This is attributable to common features of the selection environments.
2. The second term is the specific response to the selection treatment, estimated from the difference between the average of replicate lines subjected to this treatment, *R*(*ij*.), and the average of all selection treatments in this assay environment, *R*(*i*..). If *i* = *j*, this is the specific direct response, which expresses local adaptation, using replicate selection lines to test its significance. If *i* ≠ *j*, it is a specific indirect response in a given assay environment. The degree of local adaptation to the *j*‐th selection environment relative to the *i*‐th assay environment is evaluated by comparing *R*(*jj*.) with *R*(*ji*.), which corresponds to the “local versus foreign” comparison of Kawecki and Ebert (2004). The cost of adaptation in the *i*‐th assay environment is evaluated by comparing *R*(*jj*.) with *R*(*ij*.), which corresponds to the “home versus away” comparison of Kawecki and Ebert (2004).
3. The third term is the deviation of the growth of each line, *R*(*ijk*), from the average of lines subjected to a particular selection treatment and assayed in a particular environment, *R*(*ij*.). Functional interference is expressed by a negative correlation among replicate lines between the deviations for a given assay environment and those for the environment of selection, given that the direct response is positive (local adaptation has evolved) and varies among lines (potentially leading to different degrees of maladaptation).

This interpretation of a reciprocal transplant assay can be summarized like this: The specific direct response expresses the degree of local adaptation, while the specific indirect response expresses the cost of adaptation, whose source can be identified from the line‐based estimates.

## Results

### Analysis of variance

The selection × assay environment interaction after 24 cycles was significant only for the linear pathway (*F* (9, 368) = 12.5, *P* < 0.001). After 42 cycles, however, the selection × assay environment interaction was highly significant in the linear pathway (*F* (9, 368) = 6.6, *P* < 0.001), the branched pathway (*F* (9, 368) = 6.3, *P* < 0.001), and the cyclical pathway (*F* (9, 368) = 7.1, *P* < 0.001).

### Direct response to selection

The general response was positive in all cases at cycle 24. It had further increased in all lines by cycle 42, at which point average growth (over all substrates, as OD) had increased by a factor of 6.6. Substrates differed widely in the magnitude of the advance: Those where the ancestor already grew well, especially fructose and raffinose, showed only modest gains of 20–58%, whereas those where the ancestor grew poorly advanced by as much as 3 order of magnitude above the ancestral value. Hence, the general response reduced the variation of growth among substrates, roughly halving the coefficient of variation from 1.64 in the ancestors to 0.59 in the selection lines at cycle 42.

**Figure 1:**
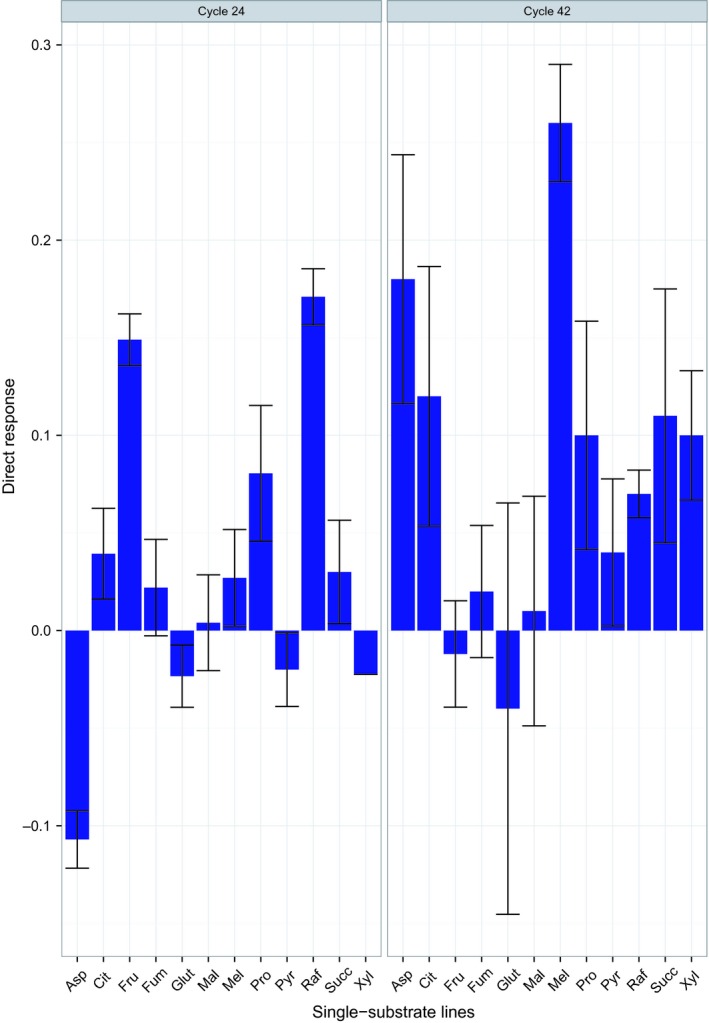
Direct response to selection as a representation for local adaptation at cycles 24 and 42. y‐Axis is the direct response to selection measured by optical density averaged over replicate lines. Error bars are the standard error of the mean.

Local adaptation is evaluated by the specific direct response, averaged over replicate lines, which was positive for 8/12 substrates at cycle 24 and for 10/12 substrates at cycle 42 (Fig. 1), with 7/10 estimates being significant at cycle 42 (*t*‐test for departure from 0, *P* < 0.05). The response also became larger (more positive) for 9/12 substrates over this interval, the exceptions being raffinose, fructose, and (marginally) fumarate. The average overall direct response was +0.029 (SE = 0.022, *t* = 1.33 for departure from 0, df = 11, *P* = 0.21) at cycle 24 and was +0.079 (SE = 0.024, *t* = 3.29 for departure from 0, df = 11, *P* < 0.01) at cycle 42. Hence, there is good evidence of local adaptation, which may have strengthened between cycles 24 and 42 (difference between average overall responses, *t* = 1.53, df = 22, *P* = 0.15). These overall growths of the lines represent advances above the growth of the ancestor of 26% at cycle 24% and 150% at cycle 42.

### Variance among lines

The variation of growth among lines when assayed in the environment of selection expresses the degree of divergence of the direct response. Estimates of the among‐line variance component were positive for all substrates and significant (*F* (11, 12) = 4.4, *P* < 0.01) for all except raffinose and xylose. The standard deviation (square root of the among‐line variance component) of the direct response increased with the mean (*r* = 0.51, df = 10, 0.05 < *P* < 0.1), showing that replicate lines diverged more in environments where growth increased more. This was attributable entirely to the specific direct response (*r* = +0.58, df = 10, *P* = 0.05) and not at all to the general response (*r* = 0.08, df = 10, *P* > 0.5).

Estimates of the among‐line variance component were positive for all combinations of different selection and assay substrates except one (glutamate assayed in xylose) and significant (*F* (11, 12) = 2.81, *P* < 0.05) for 25/36 combinations.

### The indirect response to on‐pathway substrates

The specific indirect response to substrates on the same metabolic pathway was on average about zero (18 estimates, mean = +0.0036, SE = 0.0104 at cycle 24; 18 estimates, mean = +0.009, SE = 0.020 at cycle 42). The sign of the observed values did not consistently correspond with prediction (Table 1). The lack of fit with prediction also applied when the three pathways were examined separately.

**Table 1:** Indirect response to selection to on‐pathway substrates

Selection substrate	Assay substrate	Predicted indirect response	Observed indirect response at cycle 24 ± Std‐error	Observed indirect response at cycle 42 ± Std‐error
Raffinose	Fructose	+	+0.215 ± 0.011	+0.0442 ± 0.007
Raffinose	Pyruvate	+	+0.0663 ± 0.012	−0.0016 ± 0.011
Fructose	Raffinose	+	+0.093 ± 0.016	+0.0744 ± 0.008
Fructose	Pyruvate	+	+0.069 ± 0.016	−0.0076 ± 0.018
Pyruvate	Raffinose	−	−0.0995 ± 0.018	−0.0441 ± 0.025
Pyruvate	Fructose	−	−0.1969 ± 0.018	−0.0207 ± 0.024
Succinate	Fumarate	+	−0.0067 ± 0.009	+0.0409 ± 0.034
Succinate	Malate	+	−0.0037 ± 0.009	+0.0475 ± 0.036
Fumarate	Succinate	+	+0.0307 ± 0.013	−0.0513 ± 0.014
Fumarate	Malate	+	+0.0168 ± 0.012	−0.0087 ± 0.03
Malate	Succinate	+	−0.0270 ± 0.013	−0.0049 ± 0.018
Malate	Fumarate	+	+0.0027 ± 0.012	−0.0381 ± 0.014
Proline	Glutamate	+	+0.0203 ± 0.01	−0.0067 ± 0.02
Proline	Citrulline	+	+0.0570 ± 0.017	−0.0028 ± 0.02
Glutamate	Proline	+	−0.0569 ± 0.009	−0.0440 ± 0.02
Glutamate	Citrulline	+	−0.0639 ± 0.008	−0.0532 ± 0.011
Citrulline	Proline	−	+0.0260 ± 0.014	+0.0512 ± 0.027
Citrulline	Glutamate	−	+0.0271 ± 0.009	+0.0895 ± 0.029

### The indirect response to off‐pathway substrates

**Figure 2:**
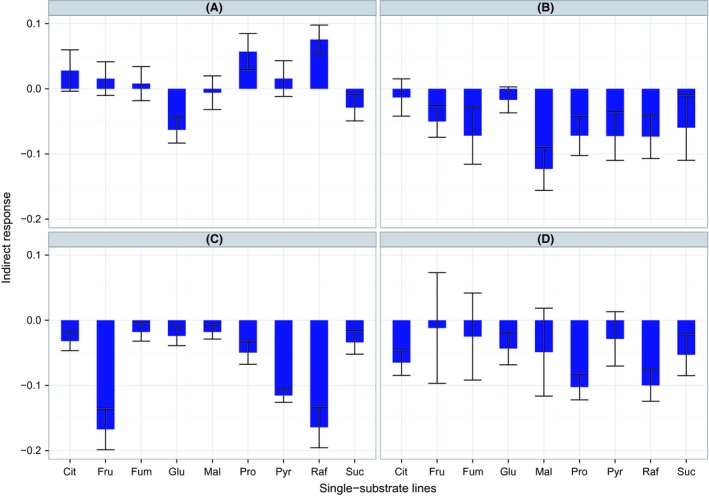
Indirect response for off‐pathway substrates (A) indirect response to off‐pathway substrates from selection on the on‐pathway substrates at cycle 24. (B) Indirect response to off‐pathway substrates from selection on the on‐pathway substrates at cycle 42. (C) Indirect response to on‐pathway substrates from selection on the off‐pathway substrates at cycle 24. (D) Indirect response to on‐pathway substrates from selection on the off‐pathway substrates at cycle 42. Error bars are standard error of the mean.

There are 18 estimates of the indirect response for off‐pathway substrates, nine from the indirect response to selection for on‐pathway substrates and nine from the indirect response to selection for the off‐pathway substrates themselves (Fig. 2A–D). About 12 of 18 were negative at cycle 24 (7/18 significant at *P* < 0.05, *t*‐test for departure from 0) and all 18 of 18 were negative at cycle 42 (mean = −0.0564, SE = 0.0074, *t* = −7.6, *P* < 0.001; 6/18 significant at *P* < 0.05, *t*‐test for departure from 0).

### The correlated response of replicate lines

Each pathway provided 12 correlations of the indirect with the direct response to selection on a given substrate. Most of these (29/36) are positive (Table 2); None of the negative correlations are significant at *P* < 0.05. There may be a tendency for off‐pathway correlations (mean = 0.282, SE = 0.102) to be somewhat lower than on‐pathway correlations (mean = 0.491, SE = 0.088; one‐tail *t*‐test; *t* = −1.63, df = 17, *P* < 0.06).

**Table 2:** Correlations of the direct with the indirect response to selection. As there are 12 comparisons for each set of substrates, the corrected *P*‐value should be considered at *P* < 0.004 (Bonferroni correction method). The bold values marked with * show statistical significance based on Bonferroni correction method (Dunn 1961)

Selection line	Assay environment	Correlation	*P*‐value
Raf	Fru	0.5987	0.0397
Raf	Pyr	−0.2854	0.3686
Raf	Asp	−0.5482	0.065
Fru	Raf	0.3235	0.305
Fru	Pyr	0.1409	0.6624
Fru	Asp	−0.1445	0.6542
Pyr	Raf	0.1277	0.6926
Pyr	Fru	−0.0466	0.8856
Pyr	Asp	0.9504	<0.0001*
Asp	Raf	0.0816	0.8009
Asp	Fru	−0.5092	0.0909
Asp	Pyr	0.9298	<0.0001*
Suc	Fum	0.9605	<0.0001*
Suc	Mal	0.9542	<0.0001*
Suc	Mel	0.5087	0.0912
Fum	Suc	0.7246	0.0077
Fum	Mal	0.9471	<0.0001*
Fum	Mel	0.4275	0.1657
Mal	Succ	0.4701	0.123
Mal	Fum	0.9133	<0.0001*
Mal	Mel	0.1264	0.6955
Mel	Succ	0.3242	0.3039
Mel	Fum	0.4106	0.1849
Mel	Mal	0.2724	0.3917
Pro	Glu	0.6035	0.0377
Pro	Cit	0.0808	0.8029
Pro	Xyl	−0.0137	0.9662
Glu	Pro	0.5596	0.0585
Glu	Cit	0.5452	0.0668
Glu	Xyl	0.3646	0.2439
Cit	Pro	0.3921	0.2074
Cit	Glu	0.813	0.0013*
Cit	Xyl	−0.0499	0.8775
Xyl	Pro	0.8659	0.0003*
Xyl	Glut	0.5091	0.091
Xyl	Cit	0.5732	0.0514

## Discussion

### The general response

The single ancestral strain was isolated from oak bark and subsequently maintained on solid agar in Petri plates using rich medium (yeast extract peptone dextrose, YPD) in the laboratory. To investigate the evolution of specialization, this strain was grown in well plates with liquid minimal medium supplemented with a single carbon substrate. Although we intended to study how lines adapted specifically to each substrate, the environment of selection had many features common to all lines and all substrates that differed from anything previously experienced by the ancestor, either in the wild or subsequently in domestication. The outcome was a rapid general response to these new conditions that was expressed regardless of the particular substrate supplied. This mirrors a more extensive experiment in which the bacterium *Pseudomonas fluorescens* was cultivated on 95 carbon substrates (MacLean and Bell 2002). When lines cultivated on a given substrate were tested on the other substrates, they grew better than the ancestor in the great majority of cases, which the authors attributed, as we do, to common environment (MacLean and Bell 2002). In both experiments, a positive general response was detected because several differential factors were used and the outcome was evaluated by a full reciprocal transplant assay. Should only a single factor be used, or should lines be tested only in the environment of selection, it would not be possible to separate the general and specific responses to selection.

### The grain of specialization

Specialization evolves through loss of function rather than through gain of function. Imagine a population of microbes in which all individuals are generalists equally capable of metabolizing a wide range of substrates. A mutation arises in a certain lineage that confers the ability to utilize one of these substrates more efficiently, and this lineage consequently displaces all of its competitors. This process does not result in specialization, however, but merely in the evolution of a more efficient generalist. The case is different if there is a trade‐off, such that the modified lineage, as the consequence of its newly acquired proficiency, loses the ability to metabolize certain other substrates, or metabolizes them less efficiently than its competitors (Reboud and Bell 1997; Cooper and Lenski 2000, 2010; Lunzer et al. 2002; MacLean and Bell 2002; Ostrowski et al. 2007; Behe 2010; Hietpas et al. 2013; Kvitek and Sherlock 2013). The population now consists of two types with somewhat different patterns of substrate metabolism and may thereafter maintain this degree of specialization through divergent natural selection.

Adaptation to a particular substrate through gain‐of‐function mutations is a direct response to selection. Any loss of adaptation to other substrates that evolves concomitantly is an indirect response to selection that constitutes a cost of adaptation. Consequently, the crucial process in the evolution of specialization is a negative indirect response to selection reflecting loss of function caused by a trade‐off of some kind (Bell 2008). In our experiment, there was a consistently negative response to off‐pathway substrates, whereas the direction of response to substrates on the same metabolic pathway was neither strong nor predictable. This suggests that the grain of specialization is the metabolic pathway, rather than the individual substrate. It is worth mentioning that the negative response to on‐pathway substrates displayed a tendency to become more negative through time (Table 1). This trend is consistent with a Fisher's geometric model of adaptation in a multipeaked landscape (Martin and Lenormand 2015; Schick et al. 2015).

This conclusion that specialization evolves among metabolic pathways is consistent with well‐established trade‐offs between major metabolic systems such as autotrophy versus heterotrophy (Reboud and Bell 1997) or fermentation versus respiration (Novak et al. 2006; Frank 2010). It is also broadly consistent with metabolic surveys of natural populations of wild yeast. Samani et al. (2015) found that wild isolates are capable of metabolizing a wide range of substrates, with no evidence for narrow specialists nor for generally negative correlations between substrates. There was, however, evidence for trade‐offs between certain categories of substrates. Growth on a dozen or so core substrates, which are metabolized efficiently by all strains, was negatively correlated with growth on ancillary substrates, whose utilization is more erratic. There was also a negative correlation between growth on hexose and pentose sugars. Experimental populations propagated on a mixture of substrates did not evolve narrow specialization to each individual substrate, but instead evolved as incomplete overlapping generalists, such that each genotype in a diverse population was capable of efficiently metabolizing many but not all of the available substrates (Barrett et al. 2005). In all these cases, neither specialization nor generalization proceeded to the limit, but rather displayed an intermediate, coarse‐grained degree of specialization.

### The source of specialization

Two replicate populations exposed to the same conditions of growth may adapt at different rates or to different extents as the result of chance events such as the order of substitution of beneficial mutations (Cooper and Lenski 2000; Burke 2012; Dettman et al. 2012; Rodríguez‐Verdugo et al. 2014). The idiosyncratic variation among replicate lines has been used to identify the cause of trade‐offs (Cooper and Lenski 2000; Funchain et al. 2000; Cooper et al. 2001; MacLean and Bell 2002). If those populations that have adapted most successfully to their new conditions are systematically the least successful in other conditions, then it can be inferred that one specialized function interferes with others. Conversely, if the degree of superiority of replicate populations in their new conditions is unrelated to their degree of inferiority in other conditions, it can be inferred that the trade‐off is caused by the effect of disuse, through the accumulation of conditionally deleterious mutations. Hence, the historical divergence of replicate lines can be used to identify the source of the trade‐off responsible for a negative specific indirect response (MacLean and Bell 2002).

Correlations between replicate lines in our experiment were generally positive, although those involving off‐pathway substrates were somewhat weaker. We conclude that selection on a single substrate will usually improve performance on other substrates in the same pathway, so that there is no trade‐off and hence no evolution of specialization at this level. Substrates on different pathways are weakly correlated or uncorrelated. The exception may prove the rule: There is a strong positive correlation (*r* > 0.9) between pyruvate and aspartate. The two may be linked, however, through the conversion of pyruvate to oxaloacetate by pyruvate carboxylase, followed by the reversible transamination of oxaloacetate to aspartate through aspartate transaminase, an anaplerotic reaction that was not anticipated when the experiment was designed. If these correlations are discounted, none of the off‐pathway comparisons are significant, and the specific indirect response to off‐pathway substrates is economically explained by mutational degradation, subject to the quantitative reservations expressed above.

This interpretation requires an adequate supply of conditionally deleterious mutations. The average population grows to about 1.5 × 10^7^ cells from an inoculum 5% as large, so each growth cycle comprised about 7.5 × 10^5^ replications. The genome size of yeast is 1.2 × 10^7^ bp, and the mutation rate per nucleotide per replication is about 3 × 10^−10^ (Lang and Murray 2008; Zhu et al. 2014). Over the 180 generations of our experiment (4.3 generations per growth cycle for 42 cycles), the total number of mutations expected per population is thus about 180 × 7.5 × 10^5^ × 1.2 × 10^7^ × 3 × 10^−10^ = 4.9 × 10^5^. In a population at equilibrium, very few, if any, of these would have yet risen to high frequency. The probability that a neutral mutation, arising once, will persist for G generations is about 2/G; as the expected number of copies any number of generations hence is unity, the mean number of individuals bearing the mutation, if it has not become extinct, is about G/2. This is only a rough argument, but a more exact treatment likewise leads to the conclusion that the abundance of a lineage descending from a unique neutral mutation cannot much exceed the number of generations elapsed since its origin (Moran 1962, p. 107). The population will thus include a modest proportion of neutral mutations, each individually at low frequency but collectively affecting a substantial proportion (from the numbers given above, about 7.5%) of the population. Our experimental populations are not at equilibrium, however, but are instead subject to strong selection. In the simplest case, a single beneficial mutation will pass to fixation. It will carry with it any neutral mutations with which it was originally associated, and as the successful lineage sweeps through the population, it will accumulate further neutral mutations. As the average population size of the lineage during this process is roughly half the total population size, the number of neutral mutations at the end of the process will be about half the number expected in a population at equilibrium. These will vary, however, from very low frequency (those arising late in the process) to near fixation (those arising early in the process). In practice, the situation may be more complicated. High‐resolution sequencing has shown that beneficial mutations spreading in evolving yeast populations are often accompanied by other mutations which do not clearly increase fitness and that sequence evolution displays a pervasive interaction between clonal interference and hitchhiking (Lang et al. 2013). There may thus be a broad spectrum of neutral mutations segregating at different frequencies in the population at any one time. Finally, the fraction of neutral mutations that are conditionally deleterious in the ancestral environment is unknown. In short, the hypothesis that conditionally deleterious mutations are responsible for loss of function in the ancestral environment is not implausible, but it has yet to be demonstrated conclusively that it is quantitatively feasible.

Specialization at the level of the metabolic pathway may have a straightforward mechanistic interpretation. The immediate target of selection is the total flux through a metabolic pathway, rather than the rate of each step in the pathway. Many pathways are controlled as a whole by a regulatory gene, or set of genes, rather than each reaction in the pathway being controlled independently. Hence, the modification of common regulatory genes may be the prevalent response to selection, at least in the short term, and this is likely to generate positive correlations among substrates in the same pathway.

## Conclusion

In our experiment, local adaptation to a given substrate did not consistently affect growth on other substrates in the same pathway. Individual lines that adapted more successfully than average tended to be superior on other substrates in the same pathway. We found no consistent evidence for trade‐offs between substrates on the same pathway. The indirect response of substrates on other pathways, however, was consistently negative, with little correlation between direct and indirect responses. We conclude that the grain of specialization in this case is the metabolic pathway and that specialization appears to evolve through mutational degradation.

## Conflict of Interest

None declared.

## References

[ece32151-bib-0001] Barrett, R. D. H. , R. C. MacLean , and G. Bell . 2005 Experimental evolution of *Pseudomonas fluorescens* in simple and complex environments. Am. Nat. 166:470–480. 16224703 10.1086/444440

[ece32151-bib-0002] Behe, M. J. 2010 Experimental evolution, loss‐of‐function mutations, and “the first rule of adaptive evolution”. Q. Rev. Biol. 85:419–445. 21243963 10.1086/656902

[ece32151-bib-0003] Bell, G. 2007 The evolution of trophic structure. Heredity (Edinb). 99:494–505. 17687253 10.1038/sj.hdy.6801032

[ece32151-bib-0202] Bell, G. 2008 Selection. The mechanism of evolution, 2nd ed Oxford Univ. Press, Oxford.

[ece32151-bib-0004] Burke, M. K. 2012 How does adaptation sweep through the genome? Insights from long‐term selection experiments. Proc. Biol. Sci. 279:5029–5038. 22833271 10.1098/rspb.2012.0799 PMC3497228

[ece32151-bib-0005] Cooper, V. S. , and R. E. Lenski . 2000 The population genetics of ecological specialization in evolving *Escherichia coli* populations. Nature 407:736–739. 11048718 10.1038/35037572

[ece32151-bib-0006] Cooper, T. F. , and R. E. Lenski . 2010 Experimental evolution with *E. coli* in diverse resource environments. I. Fluctuating environments promote divergence of replicate populations. BMC Evol. Biol. 10:11. 20070898 10.1186/1471-2148-10-11 PMC2827396

[ece32151-bib-0007] Cooper, V. S. , D. Schneider , M. Blot , and R. E. Lenski . 2001 Mechanisms causing rapid and parallel losses of ribose catabolism in evolving populations of *Escherichia coli* B. J. Bacteriol. 183:2834–2841. 11292803 10.1128/JB.183.9.2834-2841.2001 PMC99500

[ece32151-bib-0008] Dettman, J. R. , N. Rodrigue , A. H. Melnyk , A. Wong , S. F. Bailey , and R. Kassen . 2012 Evolutionary insight from whole‐genome sequencing of experimentally evolved microbes. Mol. Ecol. 21:2058–2077. 22332770 10.1111/j.1365-294X.2012.05484.x

[ece32151-bib-0201] Dunn, Olive Jean . 1961 Multiple Comparisons Among Means. J. Am. Stat. Assoc. 56:52–64.

[ece32151-bib-0009] Elena, S. F. , and R. E. Lenski . 2003 Evolution experiments with microorganisms: the dynamics and genetic bases of adaptation. Nat. Rev. Genet. 4:457–469. 12776215 10.1038/nrg1088

[ece32151-bib-0010] Frank, S. A. 2010 The trade‐off between rate and yield in the design of microbial metabolism. J. Evol. Biol. 23:609–613. 20487133 10.1111/j.1420-9101.2010.01930.x

[ece32151-bib-0011] Funchain, P. , A. Yeung , J. L. Stewart , R. Lin , M. M. Slupska , and J. H. Miller . 2000 The consequences of growth of a mutator strain of *Escherichia coli* as measured by loss of function among multiple gene targets and loss of fitness. Genetics 154:959–970. 10757746 10.1093/genetics/154.3.959 PMC1461004

[ece32151-bib-0012] Gilchrist, G. W. 1995 Specialists and generalists in changing environments. I. Fitness landscapes of thermal sensitivity. Am. Nat. 146:252–270. The University of Chicago Press for the American Society of Naturalists.

[ece32151-bib-0014] Hietpas, R. T. , C. Bank , J. D. Jensen , and D. N. A. Bolon . 2013 Shifting fitness landscapes in response to altered environments. Evolution 67:3512–3522. 24299404 10.1111/evo.12207 PMC3855258

[ece32151-bib-0015] Kassen, R. 2002 The experimental evolution of specialists, generalists, and the maintenance of diversity. J. Evol. Biol. 15:173–190.

[ece32151-bib-0016] Kassen, R. 2009 Toward a general theory of adaptive radiation: insights from microbial experimental evolution. Ann. N. Y. Acad. Sci. 1168:3–22. 19566701 10.1111/j.1749-6632.2009.04574.x

[ece32151-bib-0017] Kawecki, T. J. . 1994 Accumulation of deleterious mutations and the evolutionary cost of being a generalist. Am. Nat. 144:833–838. The University of Chicago Press.

[ece32151-bib-0018] Kawecki, T. J. , and D. Ebert . 2004 Conceptual issues in local adaptation. Ecol. Lett. 7:1225–1241.

[ece32151-bib-0019] Kawecki, T. J. , R. E. Lenski , D. Ebert , B. Hollis , I. Olivieri , and M. C. Whitlock . 2012 Experimental evolution. Trends Ecol. Evol. 27:547–560. 22819306 10.1016/j.tree.2012.06.001

[ece32151-bib-0020] Kvitek, D. J. , and G. Sherlock . 2013 Whole genome, whole population sequencing reveals that loss of signaling networks is the major adaptive strategy in a constant environment. PLoS Genet. 9:e1003972. 24278038 10.1371/journal.pgen.1003972 PMC3836717

[ece32151-bib-0021] Lang, G. I. , and A. W. Murray . 2008 Estimating the per‐base‐pair mutation rate in the yeast *Saccharomyces cerevisiae* . Genetics 178:67–82. 18202359 10.1534/genetics.107.071506 PMC2206112

[ece32151-bib-0022] Lang, G. I. , D. P. Rice , M. J. Hickman , E. Sodergren , G. M. Weinstock , D. Botstein , et al. 2013 Pervasive genetic hitchhiking and clonal interference in forty evolving yeast populations. Nature 500:571–574. 23873039 10.1038/nature12344 PMC3758440

[ece32151-bib-0023] Levins, R. 1968 Evolution in changing environments: some theoretical explorations. Princeton Univ. Press, Princeton, NJ.

[ece32151-bib-0024] Lunzer, M. , A. Natarajan , D. E. Dykhuizen , and A. M. Dean . 2002 Enzyme kinetics, substitutable resources and competition: from biochemistry to frequency‐dependent selection in lac. Genetics 162:485–499. 12242256 10.1093/genetics/162.1.485 PMC1462262

[ece32151-bib-0025] MacLean, R. C. , and G. Bell . 2002 Experimental adaptive radiation in *Pseudomonas* . Am. Nat. 160:569–581. 18707508 10.1086/342816

[ece32151-bib-0026] Martin, G. , and T. Lenormand . 2015 The fitness effect of mutations across environments: Fisher's geometrical model with multiple optima. Evolution 69:1433–1447. 25908434 10.1111/evo.12671

[ece32151-bib-0027] Meijden, E. 1996 Plant defence, an evolutionary dilemma: contrasting effects of (specialist and generalist) herbivores and natural enemies. Entomol. Exp. Appl. 80:307–310.

[ece32151-bib-0028] Moran, P. A. P. 1962 The statistical processes of evolutionary theory. Clarendon Press, Oxford.

[ece32151-bib-0029] Novak, M. , T. Pfeiffer , R. E. Lenski , U. Sauer , and S. Bonhoeffer . 2006 Experimental tests for an evolutionary trade‐off between growth rate and yield in *E. coli* . Am. Nat. 168:242–251. 16874633 10.1086/506527

[ece32151-bib-0030] Ostrowski, E. A. , C. Ofria , and R. E. Lenski . 2007 Ecological specialization and adaptive decay in digital organisms. Am. Nat. 169:E1–E20. 17206577 10.1086/510211

[ece32151-bib-0031] Reboud, X. , and Bell G. . 1997 Experimental evolution in Chlamydomonas. III. Evolution of specialist and generalist types in environments that vary in space and time. Heredity (Edinb). 78:507–514. Nature Publishing Group.

[ece32151-bib-0032] Rodríguez‐Verdugo, A. , D. Carrillo‐Cisneros , A. González‐González , B. S. Gaut , and A. F. Bennett . 2014 Different tradeoffs result from alternate genetic adaptations to a common environment. Proc. Natl Acad. Sci. USA 111:12121–12126. 25092325 10.1073/pnas.1406886111 PMC4143048

[ece32151-bib-0033] Samani, P. , E. Low‐Decarie , K. McKelvey , T. Bell , A. Burt , V. Koufopanou , et al. 2015 Metabolic variation in natural populations of wild yeast. Ecol. Evol. 5:722–732. 25691993 10.1002/ece3.1376 PMC4328774

[ece32151-bib-0034] Schick, A. , S. F. Bailey , and R. Kassen . 2015 Evolution of fitness trade‐offs in locally adapted populations of *Pseudomonas fluorescens* . Am. Nat. 186(Suppl):S48–S59, University of Chicago Press, Chicago, IL. 26656216 10.1086/682932

[ece32151-bib-0035] Stearns, S. C. . 1989 Trade‐offs in life‐history evolution. Funct. Ecol. 3:259–268. British Ecological Society.

[ece32151-bib-0036] Wei, X. , K. Vrieling , P. P. J. Mulder , and P. G. L. Klinkhamer . 2015 Testing the generalist‐specialist dilemma: the role of pyrrolizidine alkaloids in resistance to invertebrate herbivores in *Jacobaea* species. J. Chem. Ecol. 41:159–167. 25666592 10.1007/s10886-015-0551-4 PMC4351440

[ece32151-bib-0037] Zhu, Y. O. , M. L. Siegal , D. W. Hall , and D. A. Petrov . 2014 Precise estimates of mutation rate and spectrum in yeast. Proc. Natl Acad. Sci. USA 111:E2310–E2318. 24847077 10.1073/pnas.1323011111 PMC4050626

